# Clinical outcomes following preimplantation genetic testing and microdissecting junction region in couples with balanced chromosome rearrangement

**DOI:** 10.1007/s10815-020-02052-6

**Published:** 2021-01-11

**Authors:** Dehua Cheng, Liang Hu, Fei Gong, Shimin Yuan, Keli Luo, Xianhong Wu, Pingyuan Xie, Changfu Lu, Guangxiu Lu, Yue-Qiu Tan, Ge Lin

**Affiliations:** 1 Reproductive and Genetic Hospital of CITIC-Xiangya, Changsha, Hunan China; 2 Institute of Reproduction and Stem Cell Engineering, School of Basic Medical Science, Central South University, Changsha, Hunan China; 3 National Engineering and Research Center of Human Stem Cells, Changsha, Hunan China; 4 NHC Key Laboratory of Human Stem Cell and Reproductive Engineering, Central South University, Changsha, Hunan China; 5 Clinical Research Center for Reproduction and Genetics in Hunan Province, Changsha, Hunan China

**Keywords:** Preimplantation genetic testing, Chromosome-balanced rearrangement, Chromosome microdissection, Next-generation sequencing, MicroSeq

## Abstract

**Purpose:**

The purpose of this study is to summarize the clinical outcomes of apparently balanced chromosome rearrangement (ABCR) carriers in preimplantation genetic testing (PGT) cycles by next-generation sequencing following microdissecting junction region (MicroSeq) to distinguish non-carrier embryos from balanced carriers.

**Methods:**

A retrospective study of 762 ABCR carrier couples who requested PGT for structural rearrangements combined with MicroSeq at the Reproductive and Genetic Hospital of CITIC-Xiangya was conducted between October 2014 and October 2019.

**Results:**

Trophectoderm biopsy was performed in 4122 blastocysts derived from 917 PGT-SR cycles and 3781 blastocysts were detected. Among the 3781 blastocysts diagnosed, 1433 (37.9%, 1433/3781) were balanced, of which 739 blastocysts were carriers (51.57%, 739/1433) and 694 blastocysts were normal (48.43%, 694/1433). Approximately 26.39% of cycles had both carrier and normal embryo transfer, and the average number of biopsied blastocysts was 6.7. In the cumulative 223 biopsied cycles with normal embryo transfer, all couples chose to transfer the normal embryos. In the 225 cycles with only carrier embryos, the couples chose to transfer the carrier embryos in 169/225 (75.11%) cycles. A total of 732 frozen embryo transfer cycles were performed, resulting in 502 clinical pregnancies. Cumulatively, 326 babies were born; all of these babies were healthy and free of any developmental issues.

**Conclusion:**

Our study provides the first evaluation of the clinical outcomes of a large sample with ABCR carrier couples undergoing the MicroSeq-PGT technique and reveals its powerful ability to distinguish between carrier and non-carrier balanced embryos.

**Supplementary Information:**

The online version contains supplementary material available at 10.1007/s10815-020-02052-6.

## Introduction

Apparently balanced chromosomal rearrangements (ABCRs) (e.g., reciprocal translocation, Robertsonian translocation, and pericentric inversion) can lead to infertility, recurrent miscarriage, and offspring with congenital disabilities [1]. Prenatal diagnosis, gamete or embryo donation, and preimplantation genetic testing for structural rearrangements (PGT-SR) can be utilized to avoid the high reproductive risk of ABCRs. However, prenatal diagnosis after natural pregnancy may result in a miscarriage rate as high as 44–50% [2]. Furthermore, termination of a pregnancy with an unbalanced chromosomal abnormality after prenatal diagnosis may cause enormous physical and emotional pain in pregnant women and their families. Gamete or embryonic donations can eliminate the genetic risk of ABCRs, but they do not maintain consanguinity between the couple and the offspring. PGT-SR is typically utilized for ABCR carrier couples with poor reproductive history or primary infertility to improve the chances of a healthy and consanguineous pregnancy [1, 3, 4]. After 1998, when fluorescence in situ hybridization was successfully applied in PGT, PGT-SR has now become the first choice for carrier couples of many ABCRs.

With the application of blastocyst biopsy, chromosome microarray (including single nucleotide polymorphism [SNP] and array genomic comparative hybridization), and whole-genome sequencing techniques in PGT, the accuracy of PGT-SR has been dramatically improved [5–7]. PGT by comprehensive chromosome screening (PGT-CCS) techniques enables routine screening for aneuploidy and fragment deletion/duplication of 24 chromosomes. Cumulative clinical data have shown that PGT-CCS can increase the pregnancy rate and reduce the miscarriage rate by avoiding the transplantation of embryos with chromosome aneuploidy. The balanced embryos diagnosed by PGT-CCS still have a 50% chance of inheriting ABCR from their parents and will face the same fertility problems as their parents do in the future.

Several studies involving the microdissecting junction region (MicroSeq)-PGT technique have reported the differentiation of embryos with completely normal karyotypes from the balanced embryos of ABCR carriers in PGT [8–14]. However, the sample sizes in these studies are not sufficient to discern the percentage of patients who will benefit from these techniques. In this study, we clarified the efficacy of MicroSeq-PGT technology by evaluating the clinical outcomes of 762 ABCR carriers who chose our improved MicroSeq-PGT method. Our results showed that MicroSeq-PGT technology is an applicable technique for patients with ABCRs to improve their clinical reproductive outcomes.

## Materials and methods

### Study samples

A total of 762 ABCR carrier couples with an average age of 29.41 years were included in the retrospective study. These couples underwent in vitro fertilization treatment and sought PGT-SR to distinguish between carrier and non-carrier embryos at the Reproductive and Genetic Hospital of CITIC-Xiangya between October 1, 2014, and October 31, 2019. All patients signed informed consent forms for assisted reproductive technology and PGT after genetic consultation. The study procedure was conducted in compliance with the principles of the Declaration of Helsinki and was approved by the Ethics Committee of the Reproduction and Genetics Hospital of CITIC-Xiangya (LL-SC-SG-2014-013).

### Genomic DNA extraction

The genomic DNA of carrier couples was extracted from peripheral blood using Qiagen Blood DNA Kits (Valencia, CA, USA) and used to further inform breakpoint flanking region linkage SNP sites identified by polymerase chain reaction (PCR) experiments.

### Preparation of metaphase chromosomes

Metaphase chromosomes were prepared from cultured lymphocytes obtained from carriers using standard techniques. Karyotypes were determined from G-banding analysis using a standard protocol according to the International System for Human Cytogenetic Nomenclature (2016).

### Chromosome microdissection

Chromosome microdissection was performed for the 762 ABCR carriers. First, 10 μL of nuclease-free water was added to the metaphase spread areas to facilitate the attachment of the dissected material [12]. Chromosome junction fragment DNA was obtained using glass needles (tip diameter < 0.5 μm) based on chromosome microdissection. Each fragment of the derived chromosome (8–10 copies) was dissected and amplified using the GenomePlex Single Cell Whole Genome Amplification Kit (Sigma-Aldrich, St. Louis, MO, USA) following the manufacturer’s protocol. The amplified products were verified by agarose gel electrophoresis and underwent next-generation sequencing (NGS).

### NGS and construction breakpoint flank linkage SNPs

Breakpoint mapping was based on parallel sequencing with a paired-end protocol and bioinformatic analysis using the Integrative Genomics Viewer. Briefly, 100 ng of amplified microdissected DNA was fragmented by enzyme digestion and purified to yield fragments of 100–500 bp. P1 adaptor oligonucleotides from Life Technologies (Rockville, MD, USA) were ligated on repaired A-tailed fragments. Fragments of approximately 150–300 bp were separated, purified, and enriched by electrophoresis and PCR cycles. Genomic libraries were prepared using the Ion Xpress Library Kit (Life Technologies). Each DNA library was then sequenced on a Life Technologies Ion Proton system with 318 chips as paired-end 200 bp reads. Image analysis and base calling were performed using the Life Technologies 460 Flow system.

The sequence data were cleaned by removing the primer sequences and then aligned to the reference genome (hg19) using the Integrative Genomics Viewer. Briefly, sequences that could not be aligned to hg19 or multiple sites of hg19 were removed. SNPs were compared using the Single Nucleotide Polymorphism Database and the 1000 Genomes Project database (http://www.1000genomes.org). SNPs with a mutation frequency of < 30% were selected as candidate breakpoint-specific SNPs. We synthesized specific primers to amplify and sequence the selected candidate SNPs of the couples. SNPs that were heterozygous in the ABCR carriers and homozygous in their normal partners were considered to be informative SNPs.

### PGT-CCS

Pituitary desensitization was performed using a long luteal gonadotropin-releasing hormone agonist protocol based on patient situations. After oocyte retrieval, all eggs were fertilized by intracytoplasmic sperm injection. All embryos were cultured to the blastocyst stage in sequential media (G1 and G2; Vitrolife, Goteborg, Sweden). On the morning of day 6, the blastocysts were scored based on the evaluation of their trophectoderm (TE) and inner cell mass morphology according to the criteria described by Gardner and Schoolcraft, with minor differences. Approximately 3–8 TE cells were aspirated using a biopsy pipette with a 30-μm internal diameter and dissected with a Zilos TK laser (Hamilton Thorne, Beverly, MA, USA). Biopsied TE cells were then used for whole genome amplification (WGA) via multiple displacement amplification with a REPLI-g Single Cell Kit (Qiagen). Then, PGT-CCS based on NGS was performed as previously described [6].

### Carrier embryo diagnosis

The informative SNPs flanking the breakpoint were amplified via PCR in the excess WGA products of euploid embryos diagnosed via PGT-CCS and then sequenced. In each couple, 4–10 breakpoint-adjacent informative SNPs were selected for subsequent linkage analysis to distinguish non-carrier embryos and carrier embryos. The embryos that were positive for informative SNPs were predicted to be carrier embryos. The embryos that were negative for informative SNPs were predicted to be non-carrier embryos.

### Blastocyst vitrification, warming, and transfer

Blastocysts were vitrified after biopsy using Kitazato vitrification solution (Kitazato Biopharma Co., Ltd., Shizuoka, Japan) and closed high-security vitrification straws (Cryo Bio System, L’Aigle, France). After warming and dilution, blastocysts were cultured in blastocyst medium for 1–2 h. The chromosomally normal blastocysts and surviving re-expanded blastocysts with high morphological grades were selected for preferential transfer. No more than two blastocysts were transferred, and a single blastocyst transfer to each patient with well-cryopreserved embryos was recommended.

### Statistical analysis

Categorical variables were presented as percentages and compared using chi-square tests. *P* < 0.05 was considered to be statistically significant. Analyses were performed using the SPSS Statistics version 18.0 software (IBM SPSS, Armonk, NY, USA).

## Results

### Patients

In this study, 762 couples were detected to be ABCR carriers by karyotype analysis through G-banding and included 531 reciprocal translocations (263 men and 268 women), 208 Robertsonian translocations (101 men and 107 women), and 23 inversions (13 men and 10 women). In each couple, only one (male or female) carried ABCR, and the other partner had a normal karyotype.

### Summary of biopsy cycle results

**Fig. 1:**
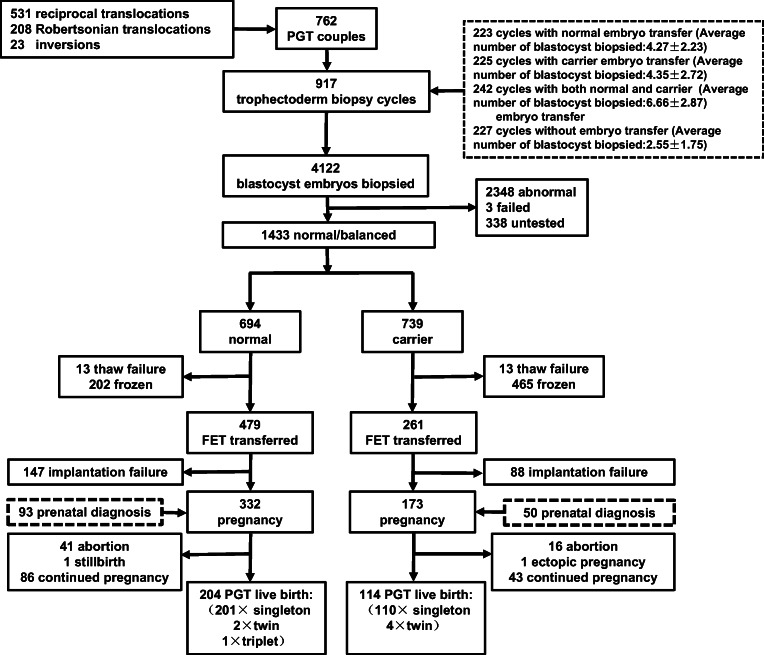
Study flow chart

In this study, TE biopsy was performed on 4122 blastocysts derived from 917 PGT-SR cycles of 762 ABCR carrier couples (618 couples undergoing one cycle, 133 couples undergoing two cycles, and 11 couples undergoing three cycles), and the average number of biopsiable blastocysts per cycle was 4.5. A total of 3781 blastocysts were detected according to the patients’ requirements (Fig. 1).

### PGT-SR results

PGT-SR was conducted on 3781 blastocysts, and the results showed that 1405 (37.16%) blastocysts were rearrangement-related unbalanced (RU) embryos, 596 (15.76%) were de novo aneuploidy (DA) embryos, 347 (9.18%) were RU + DA embryos, and 1433 (37.91%) were chromosome-balanced embryos (Table 1). Then, PGT-SR followed by ancestry-informative SNP (AISNP) linkage analysis revealed that among these balanced embryos, 694 (48.43%) were normal embryos, and 739 (51.57%) were ABCR carrier embryos.

**Table 1:** Embryo PGT results

Population	No. of embryos	RU rate, %	DA rate, %	RU + DA rate, %	Total abnormality rate, %	Non-carrier embryos rate, %	Carrier embryos rate, %
Total	3781	37.16	15.76	9.18	62.1	18.36	19.54
ROB	997	23.87	21.36	6.02	51.26	23.27	25.48
RT	2675	42.02	14.47	10.43	66.92	16.07	17.01
INV	109	29.36	8.26	5.51	43.12	29.36	27.52

*ROB* Robertsonian translocation, *RT* reciprocal translocation, *INV* inversion, *RU* rearrangement-related unbalanced, *DA* de novo aneuploidy (including unexpected abnormal fragments)

**Fig. 2:**
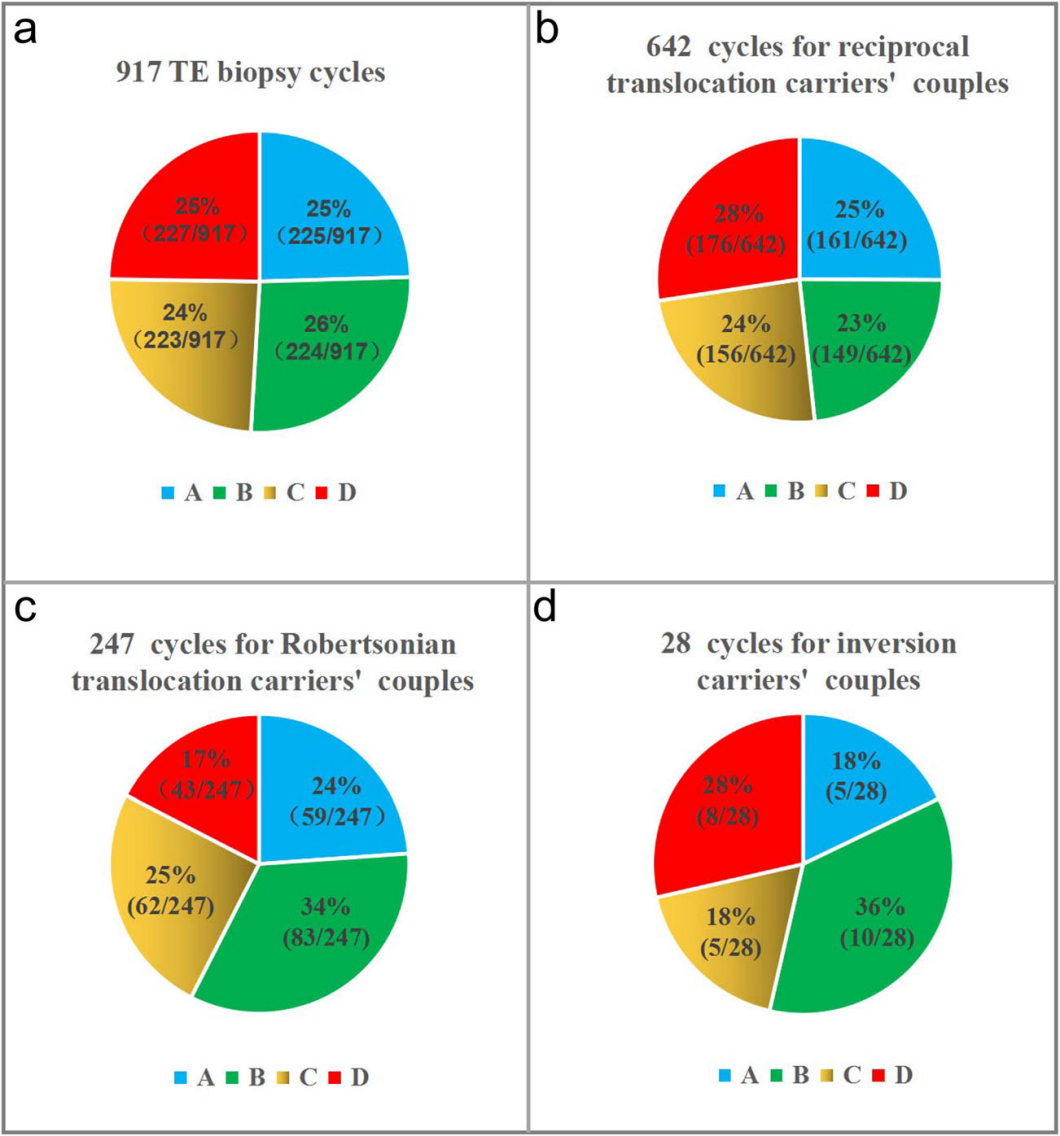
The percentages of groups **a** (only carrier embryo transfer), **b** (both carrier and normal embryo transfer), **c** (only normal embryo transfer), and **d** (without transferrable chromosome-balanced embryos) in the 917 total biopsied cycles, including 642 cycles from reciprocal translocation carrier couples, 247 cycles from Robertsonian translocation carrier couples, and 28 cycles from inversion carrier couples

**Fig. 3:**
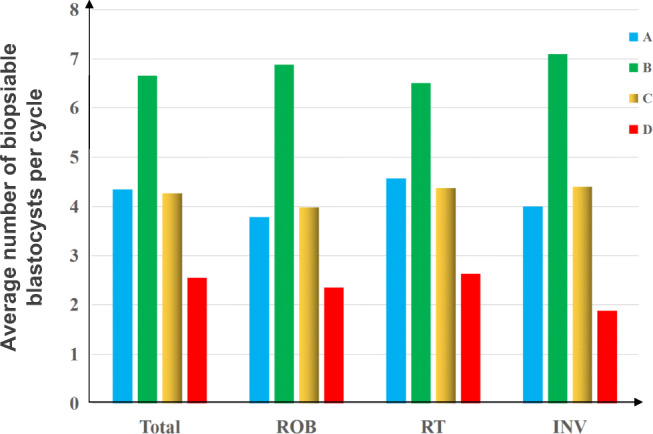
The average number of biopsiable blastocysts per cycle in groups A (only carrier embryo transfer), B (both carrier and normal embryo transfer), C (only normal embryo transfer), and D (without transferrable chromosome-balanced embryos) in the 917 total biopsied cycles, including 642 cycles from reciprocal translocation carrier couples (RT), 247 cycles from Robertsonian translocation carrier couples (ROB), and 28 cycles for inversion carrier couples (INV)

To clarify the odds of ABCR impacts on carrier and non-carrier embryo discrimination, the biopsied cycles were divided into four categories according to the results of PGT-SR: only carrier embryo transfer (group A), both carrier and normal embryo transfer (group B), only normal embryo transfer (group C), and without transferrable balanced embryos (group D). The percentages of groups A, B, C, and D in the 917 biopsied cycles were 24.54% (225), 26.39% (242), 24.32% (223), and 24.75% (227), respectively (Fig. 2a). There were no significant differences among the four groups. The proportion of biopsied cycles with normal embryos, including groups B and C, was 50.71% (465). Furthermore, in reciprocal, Robertsonian, and inversion carrier couples, the proportion of group B was 23% (149/642), 34% (83/247), and 36% (10/28), respectively, and the proportion of group C was 24% (156/642), 25% (62/247), and 18% (5/28), respectively (Fig. 2b, c, and d). Additionally, the average number of biopsied blastocysts per cycle in groups A, B, C, and D was 4.3, 6.7, 4.4, and 2.6, respectively (Fig. 3).

### Summary of clinical outcomes after frozen embryo transfer

In the cumulative 465 biopsied cycles with normal embryo transfer (groups B and C), all couples chose to transfer normal embryos. In 169 (75.11%) out of 225 cycles with only carrier embryos, the couples chose to transfer carrier embryos. A total of 479 normal embryos and 261 ABCR carrier embryos were transferred to 606 couples, resulting in 502 clinical pregnancies. So far, a cumulative total of 326 babies have been born (Fig. 1).

Between the transferred normal embryos and carrier embryos, there was no significant difference in implantation rate (normal embryo vs. carrier embryo, 332/479 [69.31%] vs. 173/261 [66.28%], *P* > 0.05), cumulative live birth rate (normal embryo vs. carrier embryo, 204/476 [42.86%] vs. 113/258 [43.80%], *P* > 0.05), and miscarriage rate (normal embryo vs. carrier embryo, 41/331 [12.39%] vs. 16/173 [9.25%], *P* > 0.05) (Table 2).

**Table 2:** Normal and carrier embryos FET outcomes

	FHB per ET, %	CLB per ET, %	Miscarriage rate, %
Normal embryos FET	69.31	42.59	12.35
Carrier embryos FET	66.28	43.68	9.25

*FET* frozen embryo transfer, *FHB* fetal heart beat, *CLB* cumulative live birth

## Discussion

Although a few studies have successfully distinguished normal embryos from carrier embryos in ABCR patients, clinical outcomes are difficult to evaluate due to the sample size limitation in these reports [14–16]. The combination of MicroSeq and AISNP analysis of ABCRs was first reported to be successfully applied in PGT-SR to discriminate normal and carrier embryos with a balanced translocation by our team in 2016 [13]. Here, we optimized this technique to make it more consistent and reproducible. Furthermore, we applied this technique to Robertsonian translocations, inversions, and reciprocal translocations. Our results showed that approximately half of the total biopsied cycles could obtain normal embryos and that the MicroSeq technique is a universal, reliable, and accurate strategy to distinguish between carrier and non-carrier balanced/euploid embryos in most patients with ABCRs.

In this study, we conducted a retrospective study of 3781 blastocysts from 917 biopsy cycles of 762 ABCR carrier couples. Our data showed that the proportion of non-carrier embryos and carrier embryos was similar in the three subgroups of different ABCR couples (Table 1). The percentage of non-carrier embryos of reciprocal translocation couples (16.07%) was lower than that of couples with Robertsonian translocation (23.27%) and inversion (29.36%). However, the possibility of bias due to small sample size in the two subgroups with Robertsonian translocation and inversion cannot be ignored. We noticed that the overall proportion of non-carrier embryos and carrier embryos in couples with reciprocal translocation was 33.08%, which was higher than the value reported previously (23.55%) [1]. These results might be valuable information in genetic and fertility counseling for these patients. Additionally, approximately 24.94% of abnormal embryos were associated with DA, reflecting the complexity of the mechanism underlying chromosomal abnormalities.

**Fig. 4:**
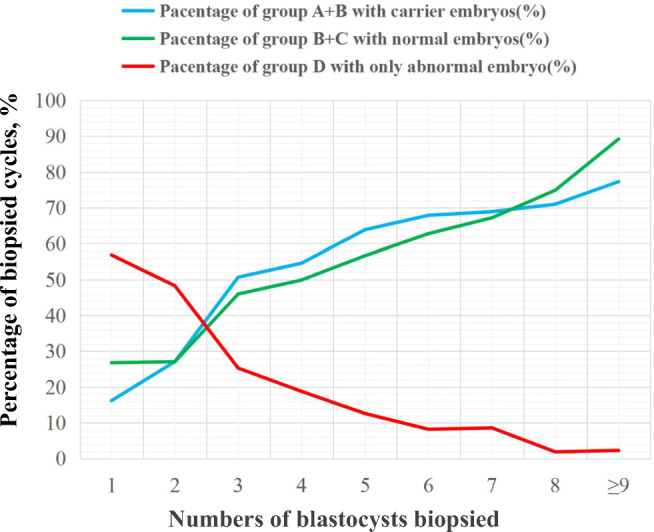
Percentage of biopsied cycles with normal embryos, carrier embryos, and only abnormal embryos in cycles with the same blastocyst numbers. Groups A (biopsied cycles with only carrier embryo transfer), B (biopsied cycles with both carrier and normal embryo transfer), C (biopsied cycles with only normal embryo transfer), and D (biopsied cycles with without transferrable chromosome-balanced embryos)

We also found that for reciprocal translocation, Robertsonian translocation, or inversion carrier couples, the average number of blastocysts in group B (biopsied cycle with both carrier and normal embryos) was significantly higher than that of the other three groups (groups A, C, and D) in a single PGT cycle. The average number of blastocysts in groups A and C was roughly equal. In addition, we observed that the percentage of cycles with normal embryos (groups B and C) was positively correlated with the number of biopsied blastocysts (Fig. 4, Supplementary Table S1). These data suggest that with the increase in the number of embryos in a single PGT cycle, ABCR couples are more likely to benefit from carrier and non-carrier embryo discrimination in PGT-SR.

In this cohort, 230 couples chose to transfer 261 ABCR carrier embryos because they did not have normal embryos in PGT-SR cycles or their normal embryos had all been used. Furthermore, we observed that in the first 762 PGT-SR cycles, 82.9% (161/194) of those couples who only had carrier embryos decided to transfer a carrier embryo. We inferred that the reasons they chose to transfer carrier embryos might include the uncertainty of the result of the next PGT-SR cycle and that carrier embryos are also within the PGT recommendation for embryo transfer. The clinical outcome of these transferred embryos showed that there was no significant difference in the implantation rate (69.31% vs. 66.28%, *P* > 0.05), miscarriage rate (12.35% vs. 9.25%, *P* > 0.05), and cumulative live birth rate (42.59% vs. 43.68%, *P* > 0.05) between the normal embryo transfer group and the carrier embryo transfer group (Table 2). Our results imply that there is no significant difference in the implantation and developmental potential of non-carrier and carrier embryos, which is consistent with a previous report [17].

Allelic dropout was observed in PCR-based PGT due to the limitation of the trace amount of DNA from biopsy samples, which influenced the accuracy of PGT [18]. Meanwhile, homologous recombination during meiosis between normal chromosomes and derivative chromosomes could also lead to misdiagnosis. To minimize this possibility, we analyzed some informative SNPs within 0–5 Mbp around the breakpoint during PGT. According to our results, homologous recombination occurred in 0.63% (9/1433) of diploid embryos, leading to uncertain results during PGT (Supplementary Table S2). However, analyzing additional adjacent SNPs may solve this problem. To date, we have recorded 100% accuracy after prenatal diagnosis in 147 fetuses.

In the current study, the MicroSeq technique was applied not only for reciprocal translocation but also for Robertsonian translocation and large fragment inversion. Our data indicate that this technique is a universal, reliable, and accurate strategy to distinguish between carrier and non-carrier balanced/euploid embryos in most patients with ABCRs. Furthermore, the MicroSeq strategy makes it possible for patients to decide whether or not to perform carrier and non-carrier embryo discrimination at any time in the PGT-SR cycle. ABCR carrier patients who have very few balanced embryos and ultimately accept carrier embryo transfer could save the cost of routine PGT-SR since the MicroSeq strategy is an option for them after PGT-SR. Furthermore, patients can also decide to perform carrier and non-carrier embryo discrimination for part of their balanced embryos to save the entire PGT cost if there are already enough normal embryos for transfer. Indeed, some of our ABCR patients benefited from this strategy, and we did not perform testing for 8.20% (338/4122) embryos.

There were some limitations to this study. Among the 1433 embryos that underwent MicroSeq and AISNP analysis, only 143 (9.98%) embryos were confirmed to be consistent with the PGT results by prenatal diagnosis. PGT results were not verified in 597 (41.66%) transferred embryos, 26 (1.81%) frozen-thawed failure embryos, and 667 (46.55%) frozen embryos, which have not been considered as transferred to date. Furthermore, the current MicroSeq technique is not suitable for identifying the breakpoints of unknown chromosomal rearrangements and small fragment inversions (≤ 20 Mb) since it is hard to recognize rearranged chromosomes and dissect the chromosome segment under a microscope. However, we do not believe that these limitations weaken the study and the potential implications of the findings, as all available prenatal diagnosis results have confirmed the accuracy of our methodology.

In summary, we reported the clinical outcomes of a large sample of ABCR carriers undergoing the MicroSeq-PGT technique and found increases in the number of biopsied embryos and the chance of obtaining normal embryos. In addition, the MicroSeq technique was validated as a clinically applicable approach for most ABCR patients to choose non-carrier embryo transfer.

## Supplementary Information

ESM 1 (DOC 27 kb)

ESM 2 (DOC 216 kb)
