# Supplementary material for: Clinical outcomes following preimplantation genetic testing and microdissecting junction region in couples with balanced chromosome rearrangement
Source: J Assist Reprod Genet. 2021 Jan 11;38(3):735–42. doi: 10.1007/s10815-020-02052-6 (PMC7910386; doi:10.1007/s10815-020-02052-6)
Supplement: Supplementary file 1 — (DOC 27 kb) [file 10815_2020_2052_MOESM1_ESM.doc]

**Supplemental Table 1 Percentage of four types (Group A\B\C\D) of PGT-SR cycles with the same number of blastocysts**

| Number of blastocysts biopsied | 1 | 2 | 3 | 4 | 5 | 6 | 7 | 8 | ≥9 |
| --- | --- | --- | --- | --- | --- | --- | --- | --- | --- |
| Pacentage of group A (%) | 16.2 | 24.5 | 28.5 | 31.1 | 30.6 | 28.8 | 24.1 | 23 | 8.3 |
| Pacentage of group B (%) | 0 | 2.6 | 22.2 | 23.5 | 33.3 | 39.1 | 44.8 | 48 | 69 |
| Pacentage of group C (%) | 26.8 | 24.5 | 23.8 | 26.4 | 23.4 | 23.7 | 22.4 | 26.9 | 20.2 |
| Pacentage of group D (%) | 56.9 | 48.3 | 25.3 | 18.8 | 12.6 | 8.2 | 8.6 | 1.9 | 2.3 |

Groups A: PGT-SR cycles only with carrier embryos transfer, Groups B: PGT-SR cycles with both carrier and normal embryos transfer, Groups C: PGT-SR cycles only with normal embryos transfer, Groups D: PGT-SR cycles without chromosomal balanced embryos.
