# Supplementary material for: Clinical outcomes following preimplantation genetic testing and microdissecting junction region in couples with balanced chromosome rearrangement
Source: J Assist Reprod Genet. 2021 Jan 11;38(3):735–42. doi: 10.1007/s10815-020-02052-6 (PMC7910386; doi:10.1007/s10815-020-02052-6)
Supplement: Supplementary file 2 — (DOC 216 kb) [file 10815_2020_2052_MOESM2_ESM.doc]

**Supplementary Table S2 Embryo list with homologous recombination at the rearrangement breakpoint region.**

| **Case** | **Rearrangement of chromosomes** | **SNP NO.** | **chr** | **snp138** | **position(hg19)** | **Ref** | **Alt** | **Carrier** | **Normal spouse** | **Embryo NO.** | | | | |
| --- | --- | --- | --- | --- | --- | --- | --- | --- | --- | --- | --- | --- | --- | --- |
|  |  |  |  |  |  |  |  |  |  | **NGS5298ZL6** |  | | | |
| MD17341 | der(11) | MD17341-S2 | chr11 | rs76581995 | 113319835 | C | A | A/C | C/C | C/C |  |  |  |  |
|  |  | MD17341-S4 | chr11 | rs116435893 | 113926143 | T | G | G/T | T/T | T/T |  |  |  |  |
|  |  | MD17341-S28 | chr11 | rs11600380 | 116670182 | T | C | C/T | T/T | T/T |  |  |  |  |
|  |  | MD17341-S14 | chr22 | rs2110412 | 21019125 | T | C | C/T | C/C | T/C |  |  |  |  |
|  | der(22) | MD17341-S21 | chr11 | rs12223674 | 117451256 | C | G | G/C | G/G | G/C |  |  |  |  |
|  |  | MD17341-S22 | chr11 | rs56703391 | 117788879 | A | T | T/A | A/A | T/A |  |  |  |  |
|  |  | MD17341-S23 | chr11 | rs78152662 | 118627219 | C | T | T/C | C/C | T/C |  |  |  |  |
|  |  |  |  |  |  |  |  |  |  | **NGS5326ZH1** |  |  |  |  |
| MD17243 | inv(5) | MD17243-S2 | chr5 | rs374228978 | 32284741 | T | C | C/T | T/T | C/T |  |  |  |  |
|  |  | MD17243-S3 | chr5 | rs142977334 | 32078310 | G | A | A/G | G/G | A/G |  |  |  |  |
|  |  | MD17243-S5 | chr5 | rs79500468 | 31413967 | C | A | A/C | C/C | A/C |  |  |  |  |
|  |  | MD17243-S6 | chr5 | rs73758184 | 31233849 | C | A | A/C | C/C | A/C |  |  |  |  |
|  |  | MD17243-S8 | chr5 | rs139845912 | 31015155 | C | T | T/C | C/C | T/C |  |  |  |  |
|  |  | MD17243-S10 | chr5 | rs28769688 | 30310472 | T | C | C/T | T/T | C/T |  |  |  |  |
|  |  | MD17243-S11 | chr5 | rs11953518 | 30227969 | T | C | C/T | T/T | C/T |  |  |  |  |
|  |  | MD17243-S15 | chr5 | rs80168978 | 172248848 | T | C | C/T | T/T | T/T |  |  |  |  |
|  |  | MD17243-S16 | chr5 | rs75582769 | 172250257 | G | A | A/G | G/G | G/G |  |  |  |  |
|  |  | MD17243-S17 | chr5 | rs113866499 | 172250658 | A | G | G/A | A/A | A/A |  |  |  |  |
|  |  | MD17243-S18 | chr5 | rs7719807 | 172344999 | C | T | T/C | C/C | C/C |  |  |  |  |
|  |  | MD17243-S20 | chr5 | rs116922427 | 172705754 | C | T | T/C | C/C | C/C |  |  |  |  |
|  |  |  |  |  |  |  |  |  |  | **NGS6169CZF** |  |  |  |  |
| MD18180 | der(4) | MD18180-S2F | chr4 | rs56898749 | 117781009 | G | A | A/G | G/G | G/G |  |  |  |  |
|  |  | MD18180-S8F | chr10 | rs57847528 | 126779618 | A | G | G/A | A/A | G/A |  |  |  |  |
|  | der(10) | MD18180-S10F | chr10 | rs12251221 | 123874077 | G | C | C/G | G/G | G/G |  |  |  |  |
|  |  | MD18180-S11F | chr10 | rs75334580 | 123981600 | C | T | T/C | T/T | C/T |  |  |  |  |
|  |  | MD18180-S11F | chr10 | rs1010897 | 123981619 | A | G | G/A | G/G | A/G |  |  |  |  |
|  |  | MD18180-S12F | chr10 | rs59999955 | 124016816 | C | G | G/C | G/G | C/G |  |  |  |  |
|  |  | MD18180-S12F | chr10 | rs11200510 | 124016914 | C | T | T/C | T/T | C/T |  |  |  |  |
|  |  | MD18180-S13F | chr10 | rs145423320 | 124728394 | C | T | T/C | C/C | C/C |  |  |  |  |
|  |  | MD18180-S17F | chr4 | rs17050196 | 119896633 | C | T | T/C | C/C | C/C |  |  |  |  |
|  |  | MD18180-S18F | chr4 | rs11938552 | 119909646 | T | C | C/T | T/T | T/T |  |  |  |  |
|  |  | MD18180-S20F | chr4 | rs117113725 | 120621812 | A | G | G/A | A/A | A/A |  |  |  |  |
|  |  |  |  |  |  |  |  |  |  | **NGS6425MLB5** |  |  |  |  |
| MD18094 | der(13;14) | MD18094-S1 | chr14 | rs1243370 | 21674214 | T | C | C/T | T/T | T/T |  |  |  |  |
|  |  | MD18094-S2 | chr14 | rs71642264 | 21458812 | G | A | A/G | G/G | G/G |  |  |  |  |
|  |  | MD18094-S5 | chr14 | rs117415219 | 21091847 | A | G | G/A | A/A | A/A |  |  |  |  |
|  |  | MD18094-S11 | chr14 | rs1780929 | 20349694 | C | T | T/C | C/C | T/C |  |  |  |  |
|  |  | MD18094-S17 | chr14 | rs1686549 | 20332855 | C | T | T/C | C/C | T/C |  |  |  |  |
|  |  | MD18094-S24 | chr13 | rs185286838 | 19658074 | T | G | G/T | T/T | T/T |  |  |  |  |
|  |  | MD18094-S12 | chr13 | rs2765198 | 19762126 | G | A | A/G | G/G | G/G |  |  |  |  |
|  |  | MD18094-S13 | chr13 | rs9551248 | 19794438 | G | A | A/G | G/G | G/G |  |  |  |  |
|  |  | MD18094-S13 | chr13 | rs631612 | 19794463 | T | C | C/T | T/T | T/T |  |  |  |  |
|  |  | MD18094-S14 | chr13 | rs4406920 | 19864904 | A | G | G/A | A/A | A/A |  |  |  |  |
|  |  |  |  |  |  |  |  |  |  | **NGS8446ZXP1** | **NGS8446ZXP2** | **NGS8446ZXP3** | **NGS8446ZXP4** | **NGS8446ZXP6** |
| MD18325 | der(13;21) | MD18325-S2 | chr21 | rs61441283 | 17791770 | A | G | G/A | A/A | A/A | A/A | A/A | G/A | G/A |
|  |  | MD18325-S4 | chr21 | rs147267739 | 17013871 | C | T | T/C | C/C | C/C | C/C | T/C | T/C | T/C |
|  |  | MD18325-S5 | chr21 | rs17241283 | 16733493 | G | A | A/G | G/G | G/G | G/G | A/G | A/G | A/G |
|  |  | MD18325-S7 | chr13 | rs4770748 | 19659081 | G | A | A/G | G/G | A/G | A/G | G/G | G/G | G/G |
|  |  | MD18325-S8 | chr13 | rs74037710 | 19705011 | T | C | C/T | T/T | C/T | C/T | T/T | T/T | T/T |
|  |  | MD18325-S9 | chr13 | rs67774360 | 20973885 | C | A | A/C | C/C | A/C | A/C | C/C | C/C | C/C |
|  |  | MD18325-S10 | chr13 | rs73160826 | 20977667 | T | G | G/T | T/T | G/T | G/T | T/T | T/T | T/T |
|  |  | MD18325-S12 | chr13 | rs9552384 | 21748844 | A | C | C/A | A/A | C/A | C/A | A/A | A/A | A/A |
|  |  | MD18325-S14 | chr13 | rs9552883 | 23805898 | T | C | C/T | T/T | C/T | C/T | T/T | T/T | T/T |
